# 

**Published:** 1861-06

**Authors:** 


### CONTENTS.

### ORIGINAL COMMUNICATIONS.

PAGE.

ARTICLE I.—Lecture Introductory to the Seventh Annual Course of Lectures, in Atlanta Medical College. By H. W. Brown, M. D., Prof. of Anatomy, .......... 565

ARTICLE II.—The Etiology and Treatment of Typhoid Eever. By V. H. Taliaferro, M. D., of Columbus, Ga., .......... 580

ARTICLE III.—The Address of Dr. Hayden Coe, .......... 592

### SELECTIONS.

On the Therapeutic Action of Bismuth .......... 618

### EDITORIAL AND MISCELLANEOUS.

To Our Patrons .......... 621

Atlanta Medical College—The War .......... 622

Medical Colleges and Journals .......... 623

New Medical College .......... 624

Woorara in Epilepsy .......... 625

An Heroic Suggestion as to the Treatment of Hydrophobia .......... 625

Vicarious Menstruation .......... 626

A Move in the Right Direction .......... 626

